# Involvement of DJ-1 in the pathogenesis of intervertebral disc degeneration via hexokinase 2-mediated mitophagy

**DOI:** 10.1038/s12276-024-01196-0

**Published:** 2024-03-27

**Authors:** Jialiang Lin, Longjie Wang, Yuhao Wu, Qian Xiang, Yongzhao Zhao, Xuanqi Zheng, Shuai Jiang, Zhuoran Sun, Dongwei Fan, Weishi Li

**Affiliations:** 1 Department of Orthopedics, Peking University Third Hospital, Beijing, China; 2 Beijing Key Laboratory of Spinal Disease Research, Beijing, China; 3 Engineering Research Center of Bone and Joint Precision Medicine, Ministry of Education, Beijing, China; 4 Peking University Health Science Center, Beijing, China; 5 The Second School of Medicine, Wenzhou Medical University, Wenzhou, China

**Keywords:** Apoptosis, Pathogenesis

## Abstract

Intervertebral disc degeneration (IDD) is an important pathological basis for degenerative spinal diseases and is involved in mitophagy dysfunction. However, the molecular mechanisms underlying mitophagy regulation in IDD remain unclear. This study aimed to clarify the role of DJ-1 in regulating mitophagy during IDD pathogenesis. Here, we showed that the mitochondrial localization of DJ-1 in nucleus pulposus cells (NPCs) first increased and then decreased in response to oxidative stress. Subsequently, loss- and gain-of-function experiments revealed that overexpression of DJ-1 in NPCs inhibited oxidative stress-induced mitochondrial dysfunction and mitochondria-dependent apoptosis, whereas knockdown of DJ-1 had the opposite effect. Mechanistically, mitochondrial translocation of DJ-1 promoted the recruitment of hexokinase 2 (HK2) to damaged mitochondria by activating Akt and subsequently Parkin-dependent mitophagy to inhibit oxidative stress-induced apoptosis in NPCs. However, silencing Parkin, reducing mitochondrial recruitment of HK2, or inhibiting Akt activation suppressed DJ-1-mediated mitophagy. Furthermore, overexpression of DJ-1 ameliorated IDD in rats through HK2-mediated mitophagy. Taken together, these findings indicate that DJ-1 promotes HK2-mediated mitophagy under oxidative stress conditions to inhibit mitochondria-dependent apoptosis in NPCs and could be a therapeutic target for IDD.

## Unlocking IDD therapy: DJ-1’s role in mitophagy regulation revealed

Low back pain, often due to intervertebral disc degeneration (IDD - a condition where the disks in the spine break down), is a common global health problem. Current treatments mainly aim at pain relief and surgery, but effective methods to slow or reverse the degeneration are lacking. This research, led by Jialiang Lin and team, investigates the role of a protein, DJ-1, in protecting nucleus pulposus cells. They conducted experiments with NPCs and discovered that DJ-1 can shield these cells from damage caused by oxidative stress by promoting mitophagy (a process that controls the number and quality of mitochondria - the cell’s energy producers). The study also found that another protein, hexokinase 2 (HK2), helps DJ-1 in this protective role. The results suggest that DJ-1 could be a potential treatment target for IDD.

This summary was initially drafted using artificial intelligence, then revised and fact-checked by the author.

## Introduction

Low back pain (LBP) affects people of almost all ages and is a major contributor to the global burden of disease^1^. Considerable evidence suggests that a significant proportion of LBP cases are caused by intervertebral disc degeneration (IDD)^2,3^. IDD is a multifactorial musculoskeletal disorder^4^. Current treatment strategies for IDD include pain relief and surgical resection^5^. However, there is a lack of clinically effective ways to reverse or delay degeneration due to the limited understanding of IDD pathogenesis.

The intervertebral disc connects two adjacent vertebrae and plays an important role in the support and movement of the spine. Structurally, the intervertebral disc consists of the inner nucleus pulposus (NP), the peripheral annulus fibrosus (AF), and cartilage endplates (CEPs) in both the upper and lower parts. Specifically, the NP is mainly composed of nucleus pulposus cells (NPCs) and the extracellular matrix (ECM). NPCs are essential for maintaining disc homeostasis through the secretion of ECM components^6^. Increased apoptosis in NPCs and the resulting imbalance in ECM metabolism are thought to play crucial roles in the pathogenesis of IDD^7–9^. Therefore, inhibiting excessive apoptosis in NPCs is expected to slow the progression of IDD.

Mitochondria are the “powerhouses” of cells and the main sites of intracellular reactive oxygen species (ROS) production. Therefore, healthy mitochondria are essential for maintaining intracellular homeostasis. However, mitochondrial dysfunction induced by various stresses leads to oxidative stress-related damage in cells and even triggers mitochondria-dependent apoptosis^10–12^. Mitophagy, which is a protective mechanism for specifically clearing damaged mitochondria, controls intracellular mitochondrial quantity and quality to maintain cellular homeostasis^13,14^. Previous studies by our group and others have demonstrated the protective role of mitophagy in IDD^15–18^. However, the molecular mechanisms of mitophagy in IDD remain to be further elucidated.

DJ-1 is a multifunctional protein encoded by the *PARK7* gene and is involved in cellular processes such as intracellular antioxidant defense^19,20^, transcriptional regulation^21^, and autophagy regulation^22^. Although DJ-1 is widely expressed and plays an important role in a variety of cells, our understanding of the function of DJ-1 is limited. Our previous study showed that DJ-1 expression was decreased in degenerating nucleus pulposus tissue and that overexpression of DJ-1 attenuated oxidative stress-induced apoptosis in NPCs^23^. These results suggest that DJ-1 has a protective effect on NPCs in IDD. A recent study revealed that DJ-1 was involved in the regulation of mitophagy in neurons^24^. However, whether DJ-1 protects against IDD by regulating mitophagy in NPCs and its underlying molecular mechanisms remain unclear.

In the present study, we found that upregulating DJ-1 attenuated oxidative stress-induced mitochondria-dependent apoptosis by promoting mitophagy in NPCs. Mechanistically, we found that DJ-1 could promote the recruitment of hexokinase 2 (HK2) to damaged mitochondria via P-S473-Akt under oxidative stress conditions, thereby activating Parkin-dependent mitophagy. In addition, the therapeutic effect of DJ-1 overexpression was examined in a rat IDD model.

## Materials and methods

### Ethics statement

All animal experiments in this study were carried out in accordance with the guidelines of the Peking University Animal Care and Use Committee. Ethics approval for this study was obtained from the Biomedical Ethics Committee of Peking University (No. LA2022421).

### Primary NPC isolation and culture

Primary rat NPCs were isolated according to previous methods^25^. The isolated NPCs were cultured in a humidified incubator at 37 °C with 5% CO_2_ in Dulbecco’s modified Eagle medium/nutrient mixture F-12 (DMEM/F12; HyClone, USA) supplemented with 10% fetal bovine serum (FBS; Gibco, USA) and 1% penicillin-streptomycin solution (Hyclone). The medium was changed every other day, and the NPCs were passaged 2–4 times. NPCs were treated with tert-butyl hydroperoxide (TBHP; Sigma‒Aldrich, USA) to induce oxidative stress in vitro.

### Lentivirus and small interfering RNA (siRNA) transfection

A lentiviral vector containing rat *Park7* (OE-*Park7*) and a lentiviral vector containing rat *Hk2* (OE-*Hk2*) were constructed by Obio Technology (China). shRNAs targeting rat *Park7* (sh*Park7*) and *Hk2* (sh*Hk2*) and a negative control shRNA (shControl) were synthesized and inserted into lentiviral vectors by Obio Technology. Transfection was performed according to the manufacturer’s instructions. For siRNA transfection, the siRNA targeting rat *Prkn* (si-Parkin) and the control siRNA (si-Control) were synthesized by RiboBio (China). siRNA transfection was performed using riboFECT™ CP transfection reagent (RiboBio, China) according to the manufacturer’s instructions.

### TUNEL assay

TUNEL staining was performed to examine apoptosis in NPCs. For NPCs seeded in the cell slides, after being fixed in 4% paraformaldehyde for approximately 1 h, NPCs were permeabilized with 0.1% Triton X-100 for 10 min. Subsequently, the cells were washed with phosphate-buffered saline (PBS) and stained with the TUNEL reaction mixture (In Situ Cell Death Detection Kit, Roche, USA) according to the manufacturer’s instructions. For NP paraffin sections, after deparaffinization and rehydration, the sections were permeabilized with 10 µg/mL proteinase K for 20 min at room temperature. Next, the sections were rinsed with PBS and stained with the TUNEL reaction mixture. In addition, 4’,6-diamidino-2-phenylindole (DAPI) was used to stain the nuclei. Finally, NPC apoptosis was assessed via fluorescence microscopy (Nikon, Japan).

### Mitochondrial membrane potential (MMP) assay

Mitochondrial membrane potential was assessed using a JC-1 MitoMP Detection Kit (Dojindo Molecular Technologies, Japan) in accordance with the manufacturer’s instructions. Briefly, treated NPCs were incubated in preformulated JC-1 working solution at 37 °C in a 5% CO_2_ incubator for 30 min, after which the supernatant was removed, and the cells were washed twice with PBS. The cells were then observed under a fluorescence microscope (Nikon) after the addition of Imaging Buffer Solution, and images were obtained. The results are shown as the fluorescence intensity ratio of red (JC-1 polymer) to green (JC-1 monomer).

### Mitophagy analysis

Mitophagy Dye (Mtphagy Dye, Dojindo Molecular Technologies) was used to assess mitophagy in treated NPCs according to the manufacturer’s instructions. Mitophagy dye is a pH-sensitive probe that localizes to mitochondria. Red puncta indicate engulfed mitochondria in an acidic lysosomal environment^26^. NPCs were incubated with 0.1 μmol/L mitophagy dye for 30 min at 37 °C prior to treatment to load the probes. Finally, the treated NPCs were observed under a fluorescence microscope (Nikon), and images were obtained.

### Protein extraction and western blot analysis

The treated NPCs were collected and lysed by radioimmunoprecipitation assay (RIPA) lysis buffer (Beyotime, China) supplemented with a protease and phosphatase inhibitor cocktail (P002, New Cell & Molecular Biotech, China). The concentrations of the extracted proteins were determined with a bicinchoninic acid (BCA) protein assay kit (Beyotime). The proteins in the cytosolic and mitochondrial fractions were separated using a cellular mitochondria isolation kit (Beyotime) according to the manufacturer’s instructions. The protein samples were separated by sodium dodecyl sulfate‒polyacrylamide gel electrophoresis (SDS‒PAGE) and subsequently transferred to polyvinylidene difluoride (PVDF) membranes (Millipore, USA). After being blocked with skim milk for 1 h, the membranes were rinsed with Tris-buffered saline containing tween 20 (TBST) and incubated with specific primary antibodies overnight at 4 °C. The membranes were then washed with TBST and incubated with HRP-conjugated secondary antibodies for 2 h at room temperature. Finally, the membranes were imaged and analyzed using an iBright CL1500 imaging system and iBright analysis software (Invitrogen, USA). The primary antibodies used were as follows: DJ-1 (#5933, Cell Signaling Technology; ab76008, Abcam), COX IV (#4850, Cell Signaling Technology), RhoGDI (#2564, Cell Signaling Technology; 10509-1-Ig, Proteintech), Cytochrome c (#11940, Cell Signaling Technology; bs-0013R, Bioss), β-actin (66009-1-Ig, Proteintech), Cleaved Caspase-3 (#9664, Cell Signaling Technology), Caspase-3 (19677-1-AP, Proteintech), Parkin (sc-32282, Santa Cruz Biotechnology), Akt (#4691, Cell Signaling Technology), P-S473-Akt (#4060, Cell Signaling Technology), Hexokinase II (HK2, #2867, Cell Signaling Technology; ab209847, Abcam), Anti-Ubiquitin Antibody (VU-0101, LifeSensors).

### Immunofluorescence staining

NPCs were seeded on cell slides (SAINING, China) and fixed with 4% paraformaldehyde for 20 min. After being rinsed with PBS, the cells were permeabilized with 0.1% Triton X-100 for 5 minutes and blocked with 10% goat serum at 37 °C for 30 min. For sections of NP, deparaffinized sections following antigen retrieval were incubated with 0.5% Triton X-100 and blocked in 10% goat serum. The sections were then incubated with specific primary antibodies overnight at 4 °C. After being rinsed with PBS, the sections were incubated with Alexa Fluor 594- or Alexa Fluor 488-conjugated secondary antibodies for 1 h at 37 °C, after which the nuclei were stained with DAPI. Finally, the cell slides or sections were observed using a fluorescence microscope (Nikon). The primary antibodies used were as follows: TOM20 (#42406, Cell Signaling Technology), LC3 (#83506, Cell Signaling Technology), Parkin (sc-32282, Santa Cruz Biotechnology), P-S473-Akt (#4060, Cell Signaling Technology), Aggrecan (13880-1-AP, Proteintech), and MMP13 (18165-1-AP, Proteintech).

### Rat IDD model

Twelve-week-old male Sprague‒Dawley rats were acquired from the Department of Laboratory Animal Science of Peking University Health Science Center. A rat tail puncture-induced IDD model was established as previously reported^27^. Briefly, after the rats were anesthetized with isoflurane, the position of the coccygeal disc (Co7/8) was determined by X-ray imaging. The skin was disinfected, and Co7/8 was punctured vertically with a 23 G needle to a controlled depth of 5 mm. The needle was then rotated 360° and held in that position for 1 minute. The adeno-associated virus (AAV) to overexpress DJ-1 (AAV-*Park7*) and downregulate HK2 (AAV-sh*Hk2*) in vivo was constructed by Obio Technology. To minimize the effect of repeated puncture on IDD, 2 μL of AAV was injected into the center of the NP through the original puncture channel using a 33 G microliter syringe. The health status of the rats was monitored daily after the surgical procedure.

### Magnetic resonance imaging (MRI)

To assess disc structure and the water content of the NP, magnetic resonance imaging was performed on the coccygeal disc of the rats at 0 and 4 weeks after the procedure using a 3.0 T MRI system (Siemens, Germany). The severity of IDD was assessed by median coronal T2-weighted images of the disks in accordance with the modified Pfirrmann grading system^28^.

### Histological analysis

Rat coccygeal disks were harvested at 4 weeks postoperatively and fixed in 4% paraformaldehyde for 48 h. After decalcification with 10% ethylenediaminetetraacetic acid (EDTA; G2520, Beijing Solarbio Science & Technology Co., Ltd., China) and dehydration, the specimens were embedded in paraffin and serially sectioned at a thickness of 5 µm. To evaluate the cellularity and morphology of the NP, AF, and CEP, hematoxylin-eosin (HE) and safranin O-fast green (SO&FG) staining were performed on the obtained sections. The stained sections were scanned with a histopathology scanner (Hamamatsu Photonics K.K., Japan), and the acquired images were scored by a group of experienced histopathology researchers according to a previously reported histological scoring system^27^. The scoring system was based on five categories of degenerative changes (primarily according to the cellularity and morphology of the NP and AF), with scores ranging from five points for a normal disc to 15 points for a severely degenerated disc.

### Statistical analysis

Statistical analyses were performed using GraphPad Prism 7.00 (GraphPad Software, USA). The data were presented as the mean ± standard deviation (SD). The normality of the data distribution was assessed using the Shapiro‒Wilk test. Student’s *t*-test (*t*-test) was used to compare two groups, and one-way analysis of variance (ANOVA) followed by Tukey’s post hoc test was used to compare more than two groups. *P* < 0.05 was considered to indicate a statistically significant difference.

## Results

### Characterization of the subcellular distribution of DJ-1 in NPCs under oxidative stress conditions

**Fig. 1:**
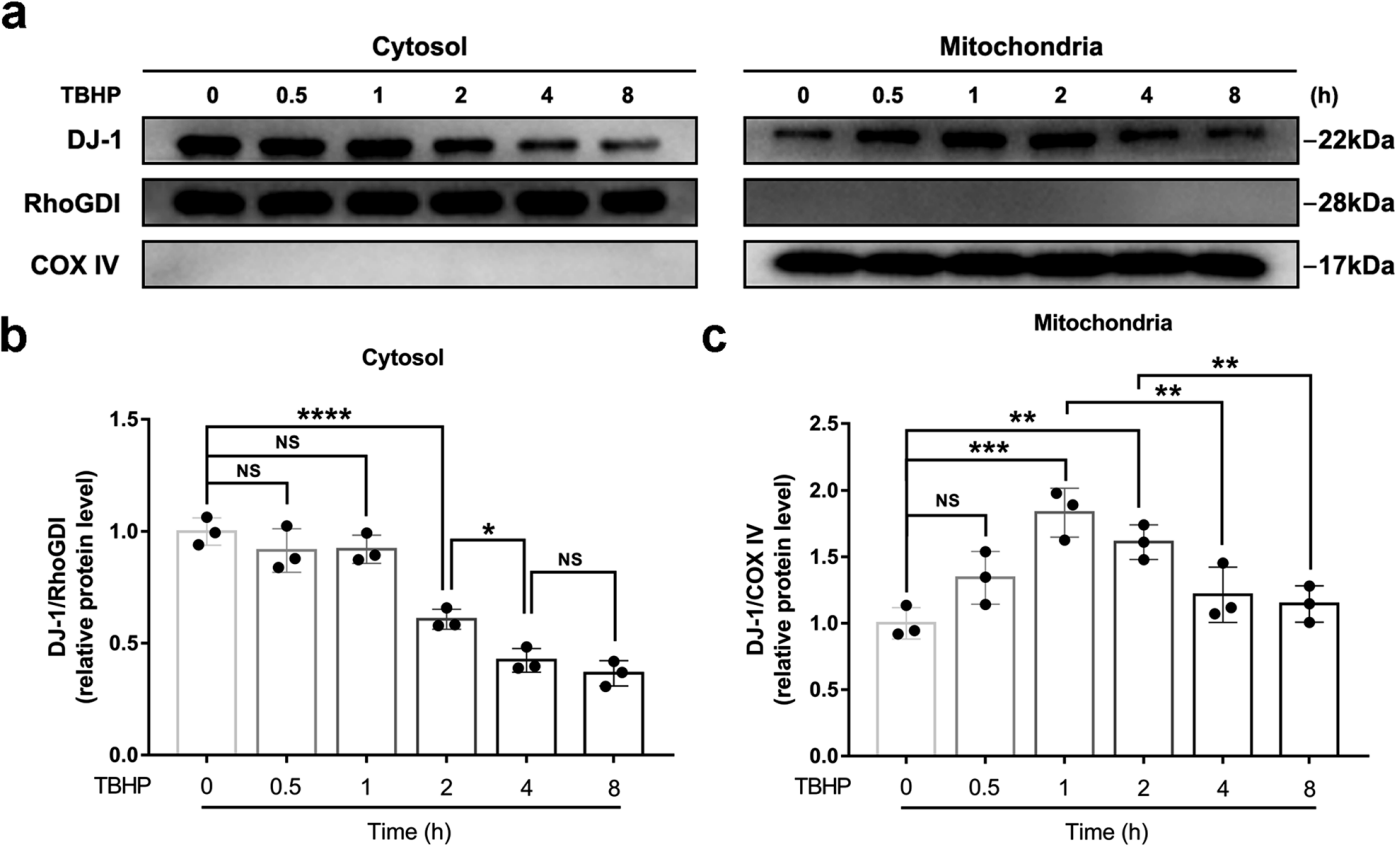
Subcellular distribution of DJ-1 in NPCs in response to oxidative stress. **a** Western blot analysis of DJ-1 in the cytosolic and mitochondrial fractions of NPCs after treatment with TBHP (50 μM) for different times. **b**, **c** Corresponding densitometric quantification of DJ-1 in the cytosolic and mitochondrial fractions. The quantitative data were presented as the mean ± SD. All the experiments were repeated independently three times. *P < 0.05; **P < 0.01; ***P < 0.001; ****P < 0.0001; NS not statistically significant.

Subcellular fractionation was performed to determine the localization of DJ-1 in NPCs under oxidative stress conditions. NPCs were treated with 50 μM TBHP for the indicated times. The western blot results showed that the DJ-1 levels in the cytosol were not significantly changed during the early stage (<2 h) of oxidative stress, while the mitochondrial localization of DJ-1 increased. Notably, the localization of DJ-1 in both the cytosol and mitochondria showed a decreasing trend during the late stage (≥2 h) of oxidative stress (Fig. 1a–c). These results suggest that DJ-1 translocates to mitochondria in NPCs during the early stage of oxidative stress.

### DJ-1 protects NPCs from oxidative stress-induced mitochondria-dependent apoptosis

**Fig. 2:**
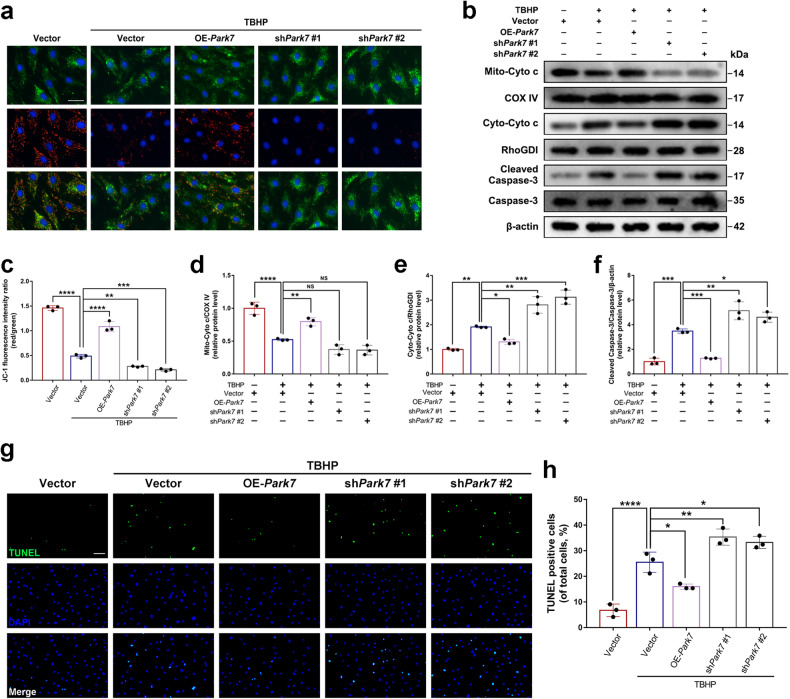
DJ-1 inhibits mitochondria-dependent apoptosis induced by oxidative stress in NPCs. Overexpression or knockdown of *Park7* by lentiviral (OE-*Park7*, sh*Park7*) transfection in primary rat NPCs prior to TBHP treatment. **a**, **c** Representative images of JC-1 staining (scale bar: 50 μm) of NPCs treated as described and the corresponding quantitative analysis. **b** Western blot analysis of Caspase-3, Cleaved Caspase-3, and Cytochrome c in the cytosol (Cyto-Cyto c) and mitochondria (Mito-Cyto c) of NPCs treated as described. **d**–**f** Densitometric analysis of Mito-Cyto c, Cyto-Cyto c, Caspase-3, and Cleaved Caspase-3. **g**, **h** TUNEL staining and quantitative analysis of the percentage of TUNEL-positive NPCs (scale bar: 50 μm). The quantitative data were presented as the mean ± SD. All the experiments were repeated independently three times. *P < 0.05; **P < 0.01; ***P < 0.001; ****P < 0.0001; NS not statistically significant.

To clarify the role of DJ-1 in mitochondrial translocation in response to oxidative stress, we first assessed mitochondrial membrane potential (MMP) by JC-1 staining. The successful overexpression and knockdown of DJ-1 in NPCs are shown in Supplementary Fig. 1. The ratio of JC-1 polymers (red) to monomers (green) reflects the level of MMP. As shown in Fig. 2a, c, TBHP-induced oxidative stress significantly decreased the ratio of JC-1 polymers to monomers. Notably, overexpression of DJ-1 significantly inhibited the TBHP-induced decrease in the ratio, while inhibition of DJ-1 expression exacerbated the decrease in the ratio. Moreover, western blot analysis of Cytochrome c in mitochondria (Mito-Cyto c) and the cytosol (Cyto-Cyto c), as well as Caspase-3, showed that overexpression of DJ-1 reduced oxidative stress-induced leakage of Cytochrome c from mitochondria to the cytosol and the subsequent activation of Caspase-3, while inhibiting DJ-1 expression exacerbated oxidative stress-induced cytosolic translocation of Cytochrome c and the activation of Caspase-3 (Fig. 2b, d–f). Furthermore, TUNEL staining was performed to assess NPC apoptosis. The results showed that the apoptotic rate of NPCs induced by oxidative stress was reduced by DJ-1 overexpression, whereas inhibiting DJ-1 expression increased the level of oxidative stress-induced apoptosis in NPCs (Fig. 2g, h). These results suggest that DJ-1 inhibits mitochondria-dependent apoptosis in NPCs under oxidative stress conditions.

### DJ-1 promotes Parkin-dependent mitophagy in NPCs

**Fig. 3:**
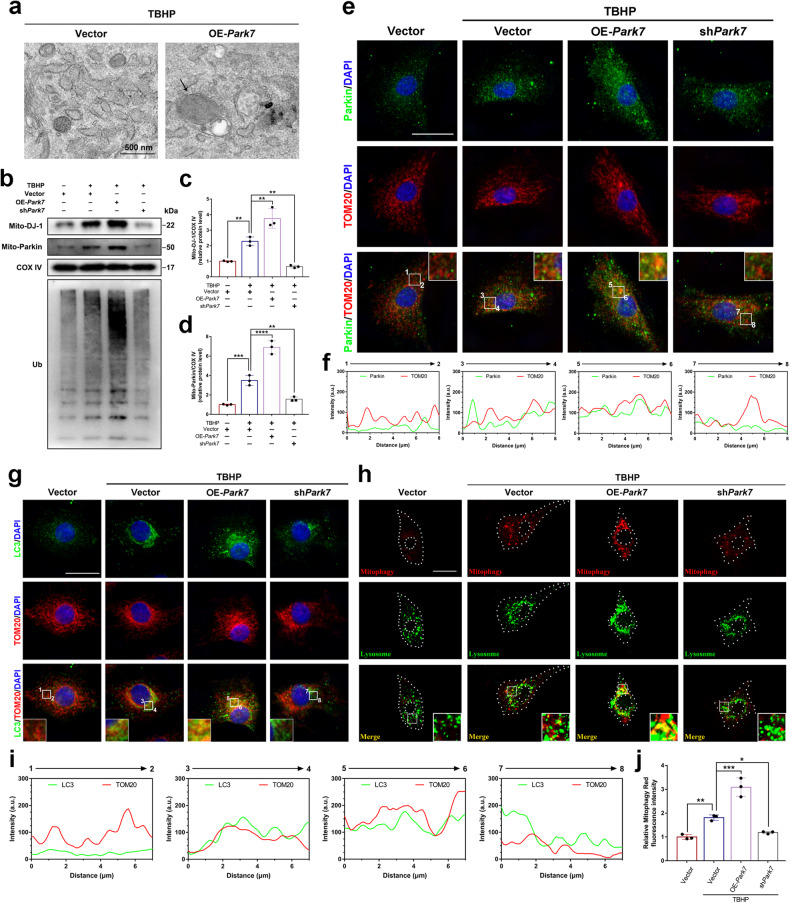
DJ-1 promotes Parkin-dependent mitophagy in NPCs. Overexpression or knockdown of *Park7* by lentiviral (OE-*Park7*, sh*Park7*) transfection in primary rat NPCs prior to TBHP treatment. **a** Representative transmission electron microscopy images of NPCs with or without OE-*Park7* transfection under oxidative stress conditions (scale bar: 500 nm). **b**–**d** Western blot and densitometric analysis of DJ-1 (Mito-DJ-1), Parkin (Mito-Parkin), and Ubiquitin (Ub) in the mitochondrial fractions of NPCs treated as described. **e**, **f** Immunofluorescence colocalization staining and analysis of Parkin and TOM20 in NPCs treated as described (scale bar: 25 μm). **g**, **i** Immunofluorescence colocalization staining and analysis of LC3 and TOM20 in NPCs as described (scale bar: 25 μm). **h** Mitophagy in NPCs was assessed by co-staining with mitophagy dye and a lysosome probe (scale bar: 25 μm). **j** Relative quantification of the mitophagy fluorescence intensity. The data are presented as the mean ± SD. All the experiments were repeated independently three times. *P < 0.05; **P < 0.01; ***P < 0.001; ****P < 0.0001.

Mitophagy is one of the most critical mechanisms for maintaining mitochondrial homeostasis in the context of cellular stress^29,30^. Previous studies have demonstrated that the proper promotion of mitophagy exerts protective effects on NPCs under oxidative stress conditions^15,31,32^. Transmission electron microscopy (TEM) was performed to observe whether DJ-1 affected mitophagy in NPCs under oxidative stress conditions. Representative TEM images showed bilayer membrane structures wrapped around damaged mitochondria in NPCs overexpressing DJ-1 (Fig. 3a). Furthermore, the western blot results showed that overexpression of DJ-1 under oxidative stress conditions increased the translocation of DJ-1 and Parkin to mitochondria, whereas inhibiting DJ-1 expression decreased the mitochondrial translocation of DJ-1 and Parkin. Notably, overexpression of DJ-1 under oxidative stress conditions promoted the ubiquitination of mitochondrial proteins, whereas inhibiting DJ-1 expression had the opposite effect (Fig. 3b–d). Furthermore, immunofluorescence colocalization staining showed that overexpression of DJ-1 under oxidative stress conditions increased the colocalization of a mitochondrial outer membrane protein (TOM20) with Parkin and the autophagosome marker LC3. Conversely, DJ-1 knockdown resulted in a significant decrease in the abovementioned colocalization (Fig. 3e–g, i). The colocalization of the fluorescent signals of mitophagy (red) and lysosomes (green) was used to examine the autophagic flux during mitophagy. As shown in Fig. 3h, j, NPCs overexpressing DJ-1 exhibited higher fluorescent intensity associated with mitophagy and more puncta that overlapped with lysosomes under oxidative stress, while NPCs with DJ-1 knockdown had the opposite effects. These results indicate that DJ-1 promotes Parkin-dependent mitophagy in NPCs under oxidative stress conditions.

### Parkin-dependent mitophagy is involved in the protective effect of DJ-1 on NPCs in response to oxidative stress

**Fig. 4:**
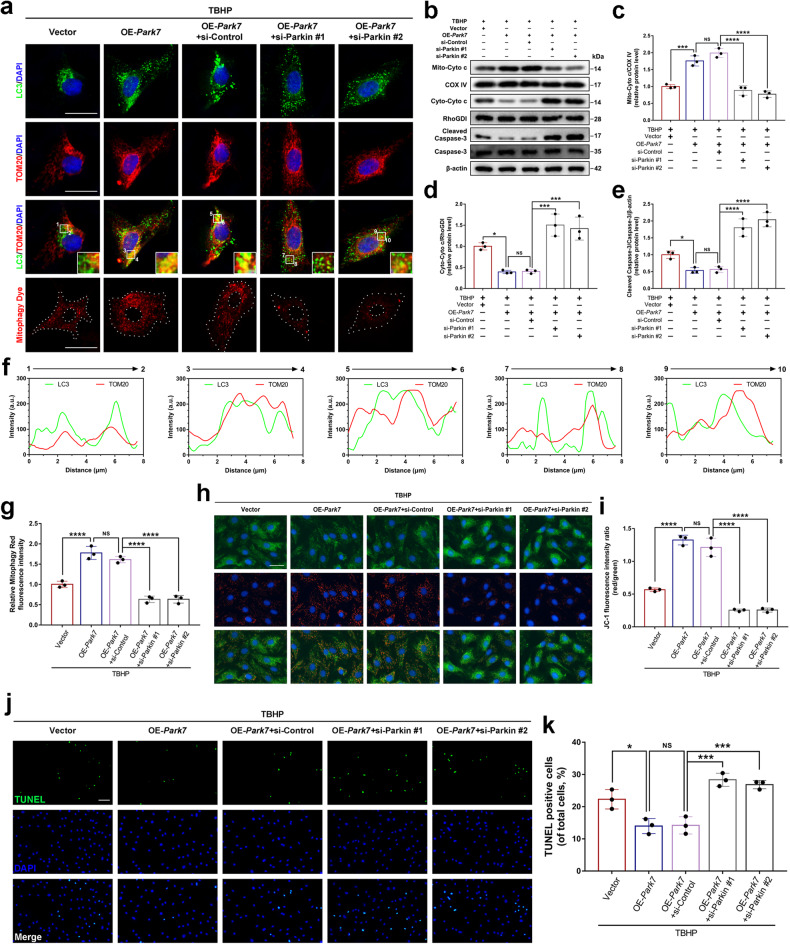
DJ-1 inhibits mitochondria-dependent apoptosis in NPCs via Parkin-dependent mitophagy in response to oxidative stress. DJ-1-overexpressing NPCs were treated with TBHP after *Prkn* was silenced with siRNA (si-Parkin). **a**, **f**, **g** Immunofluorescence colocalization staining and analysis of LC3 and TOM20 in NPCs treated as described above, as well as mitophagy staining and relative quantification of the fluorescence intensity (scale bar: 25 μm). **b**–**e** Western blot and densitometric analysis of Mito-Cyto c, Cyto-Cyto c, Cleaved Caspase-3, and Caspase-3 in NPCs treated as described above. **h**, **i** Representative images of JC-1 staining (scale bar: 50 μm) of NPCs treated as described above and the corresponding quantitative analysis. **j**, **k** TUNEL staining and quantitative analysis of the percentage of TUNEL-positive NPCs (scale bar: 50 μm). The quantitative data were presented as the mean ± SD. All the experiments were repeated independently three times. *P < 0.05; ***P < 0.001; ****P < 0.0001; NS not statistically significant.

To determine whether the anti-apoptotic effect of DJ-1 was associated with Parkin-dependent mitophagy, Parkin was knocked down in NPCs via siRNA transfection. Immunofluorescence analysis revealed that siRNA-mediated Parkin knockdown resulted in a reduction of the LC3 and TOM20 colocalization dots in DJ-1-overexpressing NPCs under oxidative stress conditions (Fig. 4a, f). Moreover, the red fluorescence intensity, which indicates mitophagy, was decreased after Parkin was knocked down in DJ-1-overexpressing NPCs (Fig. 4a, g). On the other hand, the ratio of JC-1 polymers to monomers decreased after the knockdown of Parkin in DJ-1-overexpressing NPCs under oxidative stress conditions (Fig. 4h, i). Furthermore, western blot analysis revealed that knockdown of Parkin in DJ-1-overexpressing NPCs increased Cytochrome c translocation from the mitochondria to the cytosol and the activation of Caspase-3 under oxidative stress conditions (Fig. 4b–e). TUNEL assays further confirmed the western blot results regarding apoptosis (Fig. 4j, k). These results indicate that the inhibition of Parkin-dependent mitophagy prevents DJ-1-mediated suppression of mitochondria-dependent apoptosis in NPCs under oxidative stress conditions.

### HK2 mediates Parkin-dependent mitophagy, which is promoted by DJ-1

**Fig. 5:**
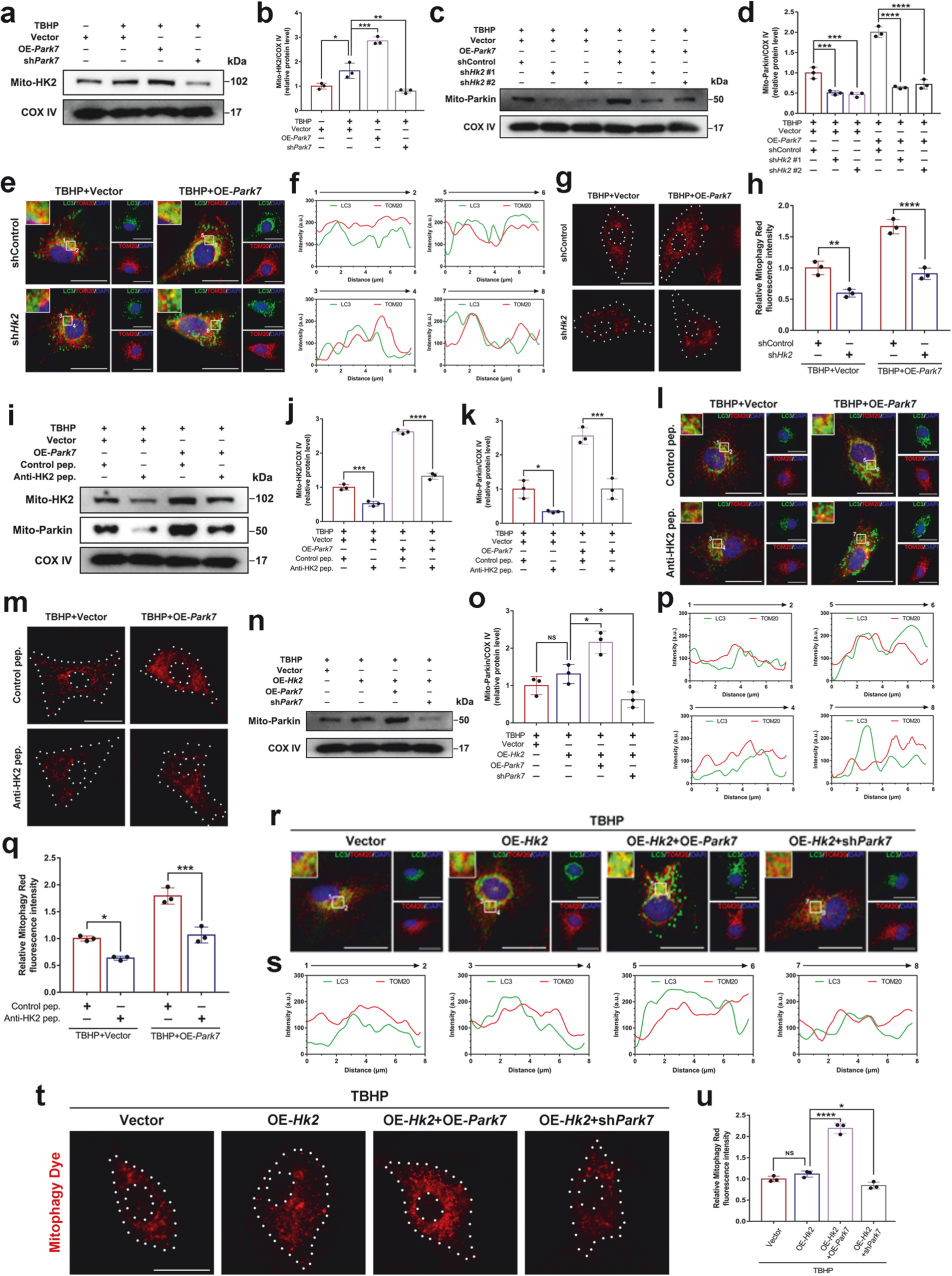
Mitochondrial translocation of HK2 is involved in DJ-1-mediated Parkin-dependent mitophagy. **a**, **b** Western blot and densitometric analysis of HK2 (Mito-HK2) in the mitochondrial fraction of NPCs transfected with OE-*Park7* or sh*Park7* under oxidative stress conditions. **c**, **d** Western blot and densitometric analysis of Mito-Parkin in DJ-1-overexpressing and control NPCs with or without *Hk2* knockdown via lentiviral (sh*Hk2*) transfection under oxidative stress conditions. **e**, **f** Immunofluorescence colocalization staining and analysis of LC3 and TOM20 in NPCs as described above (scale bar: 25 μm). **g**, **h** Mitophagy staining and relative quantification of the fluorescence intensity of NPCs as described above (scale bar: 25 μm). **i**–**k** Western blot and densitometric analysis of Mito-HK2 and Mito-Parkin in DJ-1-overexpressing and control NPCs treated with the control cell-permeable peptide (Control pep.) or the anti-HK2 cell-permeable peptide (Anti-HK2 pep.) under oxidative stress conditions. **l**, **p**. Immunofluorescence colocalization staining and analysis of LC3 and TOM20 in NPCs as described above (scale bar: 25 μm). **m**, **q** Mitophagy staining and relative quantification of the fluorescence intensity of NPCs as described above (scale bar: 25 μm). **n**, **o** Western blot and densitometric analysis of Mito-Parkin in HK2-overexpressing (OE-*Hk2*) NPCs transfected with OE-*Park7* or sh*Park7* under oxidative stress conditions. **r**, **s** Immunofluorescence colocalization staining and analysis of LC3 and TOM20 in NPCs as described above (scale bar: 25 μm). **t**, **u** Mitophagy staining and relative quantification of the fluorescence intensity of NPCs as described above (scale bar: 25 μm). The data were presented as the mean ± SD. All the experiments were repeated independently three times. *P < 0.05; **P < 0.01; ***P < 0.001; ****P < 0.0001; NS not statistically significant.

Previous studies have reported that HK2 can protect against mitochondria-dependent apoptosis^33,34^, and HK2 may be a key component of the “mitochondrial-autophagosome synapse” that promotes Parkin-dependent mitophagy^35^. To investigate whether HK2 is involved in DJ-1-induced mitophagy, western blotting was performed to examine mitochondrial recruitment of HK2. DJ-1 overexpression increased the mitochondrial localization of HK2 (Mito-HK2) under oxidative stress conditions, while DJ-1 knockdown inhibited the mitochondrial translocation of HK2 (Fig. 5a, b). The successful overexpression and knockdown of HK2 in NPCs were validated, as shown in Supplementary Fig. 2. Furthermore, western blot analysis revealed that HK2 knockdown via shRNA in control and DJ-1-overexpressing NPCs reduced mitochondrial recruitment of Parkin under oxidative stress conditions (Fig. 5c, d). Subsequently, immunofluorescence colocalization analysis and mitophagy staining showed that HK2 knockdown reduced the colocalized dots of TOM20-LC3 and the fluorescent intensity of mitophagy under oxidative stress conditions (Fig. 5e–h). To further validate the role of HK2 in DJ-1-induced mitophagy, a cell-permeable peptide (anti-HK2 pep.) that competitively binds the mitochondrial binding motif of HK2 was designed to antagonize the mitochondrial translocation of HK2^36^. The western blot results showed that anti-HK2 pep. reduced the recruitment of HK2 and Parkin to mitochondria in control and DJ-1-overexpressing NPCs under oxidative stress conditions (Fig. 5i–k). Further immunofluorescence analysis and mitophagy staining revealed that anti-HK2 pep. reduced the number of colocalized TOM20-LC3 puncta and the intensity of mitophagy in the context of oxidative stress (Fig. 5l, m, p, q). These results demonstrate that the promotion of Parkin-dependent mitophagy by DJ-1 is mediated by HK2.

Notably, the overexpression of HK2 alone in NPCs under oxidative stress failed to increase mitochondrial recruitment of Parkin or subsequent mitophagy. However, upregulation of DJ-1 expression in HK2-overexpressing NPCs increased mitochondrial recruitment of Parkin and mitophagic flux, while knockdown of DJ-1 inhibited mitochondrial recruitment of Parkin and mitophagy flux (Fig. 5n, o, r–u).

### DJ-1-induced mitophagy mediated by HK2 ameliorates IDD in rats

**Fig. 6:**
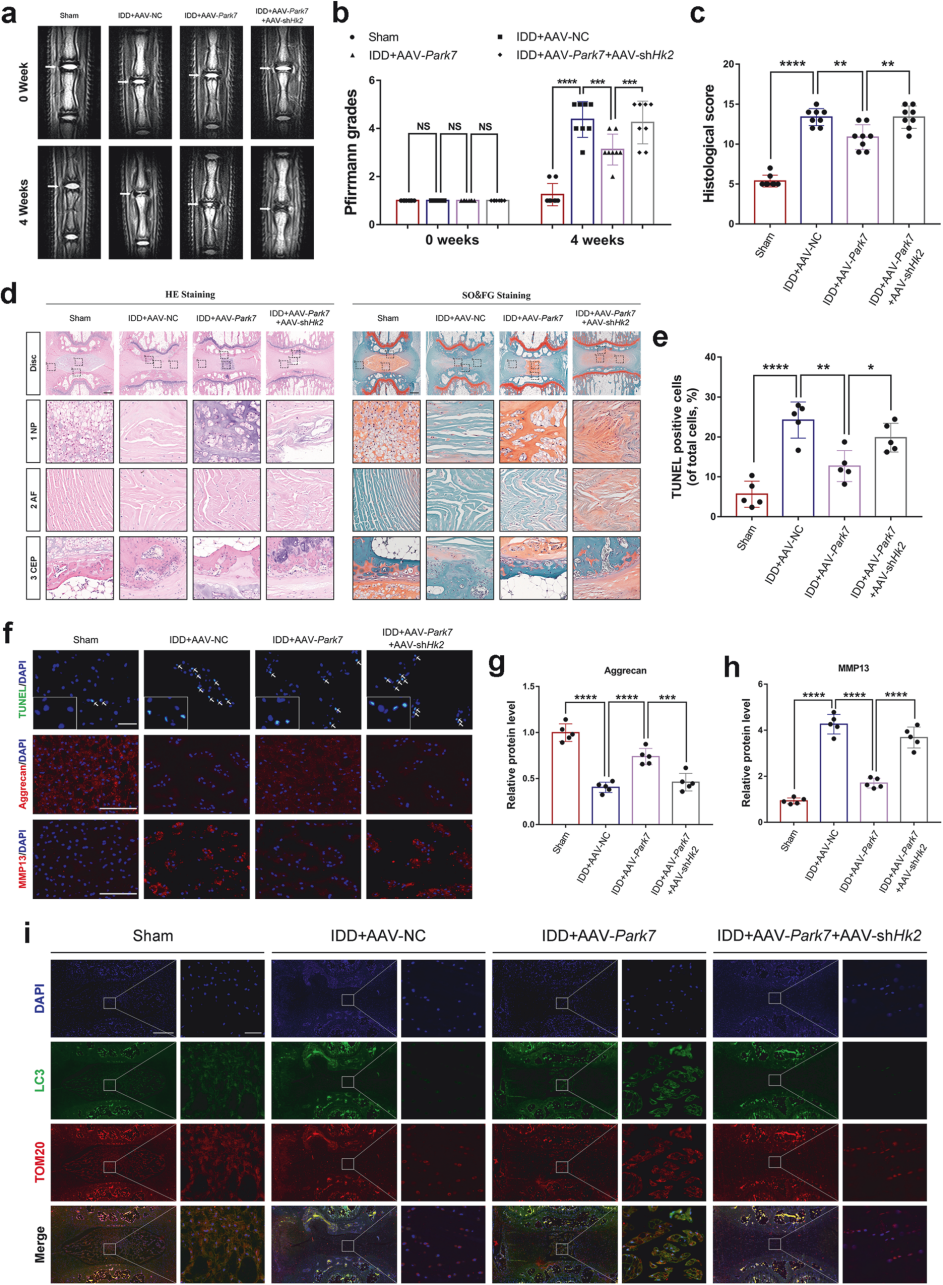
DJ-1-induced mitophagy mediated by HK2 ameliorates IDD in rats. **a**, **b** Representative median coronal T2-weighted MRI images and Pfirrmann grading scores of the coccygeal disc (white arrows) in rats at 0 and 4 weeks postsurgery. *n* = 8. **c**, **d** Histological scores and representative images of hematoxylin-eosin (HE) and safranin O-fast green (SO&FG) staining of intervertebral disks in the four groups at 4 weeks postsurgery (scale bar: 500 μm). *n* = 8. **e**–**h** TUNEL staining (scale bar: 50 μm) and quantification of the percentage of TUNEL-positive cells in NP tissue sections at 4 weeks postsurgery, as well as immunofluorescence staining of Aggrecan and MMP13 (scale bar: 100 μm) and the relative quantification of the fluorescence intensity. *n* = 5. **i** Immunofluorescence colocalization (original images, scale bar: 500 μm; enlarged images, scale bar: 5 μm) of LC3 and TOM20 in NP tissue sections at 4 weeks postsurgery. The data were presented as the mean ± SD. *P < 0.05; **P < 0.01; ***P < 0.001; ****P < 0.0001; NS not statistically significant.

To further evaluate the therapeutic effect and molecular mechanism of DJ-1 on IDD in vivo, the rat tail puncture-induced IDD model was established. The severity of coccygeal disc degeneration was first assessed by MRI at 0 and 4 weeks after the puncture procedure. As shown in Fig. 6a, b, the median coronal T2-weighted images at 0 weeks had high signal intensity, and there were no differences in the Pfirrmann grades between the groups. After 4 weeks, the IDD + AAV-*Park7* group had a higher signal intensity and lower Pfirrmann grade than the IDD + AAV-NC group. However, the IDD + AAV-*Park7* + AAV-sh*Hk2* group exhibited a lower signal intensity and higher Pfirrmann grade than the IDD + AAV-*Park7* group. Furthermore, HE and SO&FG staining showed almost disappearance of NPCs in the NP tissues of the IDD + AAV-NC group, which was accompanied by serpentine fibers in the AF and destruction of the CEP. In contrast, AAV-*Park7* treatment ameliorated the degenerative phenotype of NP, AF, and CEP tissues. However, the improvement in IDD induced by AAV-*Park7* was counteracted by AAV-sh*Hk2* (Fig. 6c, d). In addition, overexpression of DJ-1 in NP tissues by AAV-*Park7* attenuated puncture-induced apoptosis of NPCs, promoted extracellular matrix anabolism, and inhibited catabolism. However, the knockdown of HK2 abolished the ameliorative effect of DJ-1 overexpression on IDD (Fig. 6e–h). Immunofluorescence colocalization staining revealed that NPCs exhibited more LC3 and TOM20 colocalization after the overexpression of DJ-1 in NP tissues than those in the IDD + AAV-NC group. However, the knockdown of HK2 reduced LC3-TOM20 colocalization in NP tissues (Fig. 6i). Taken together, these results suggest that DJ-1 overexpression ameliorates puncture-induced IDD in rats by promoting mitophagy, which is mediated by HK2.

### DJ-1 and HK2 may be linked by activated Akt

**Fig. 7:**
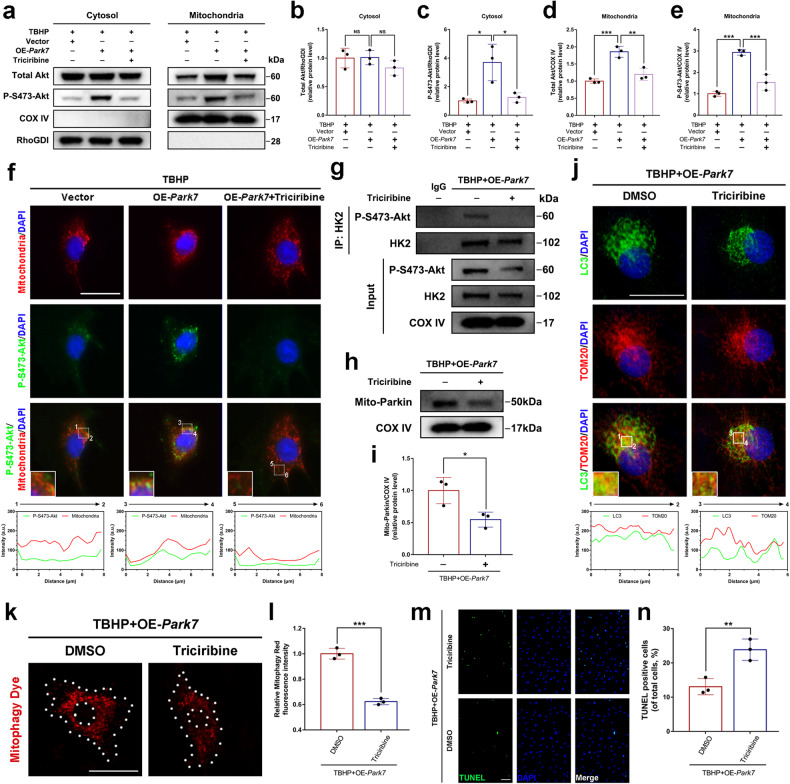
Activated Akt may link DJ-1 to HK2. DJ-1-overexpressing NPCs were treated with TBHP after being treated with triciribine. **a**–**e** Western blot and densitometric analysis of total Akt and P-S473-Akt in the cytosolic and mitochondrial fractions of NPCs treated as indicated. **f** Immunofluorescence colocalization staining and analysis of mitochondria and P-S473-Akt in NPCs treated as described above (scale bar: 25 μm). **g** Mitochondrial lysates from the indicated groups were immunoprecipitated with an anti-HK2 antibody, and western blotting was subsequently performed to measure the levels of P-S473-Akt and HK2. **h**, **i** Western blot and densitometric analysis of Mito-Parkin in DJ-1-overexpressing NPCs treated with DMSO or triciribine under oxidative stress conditions. **j** Immunofluorescence colocalization staining and analysis of LC3 and TOM20 in DJ-1-overexpressing NPCs treated as described above (scale bar: 25 μm). **k**, **l** Mitophagy staining and relative quantification of the fluorescence intensity of DJ-1-overexpressing NPCs treated as described above (scale bar: 25 μm). **m**, **n** TUNEL staining and quantitative analysis of the percentage of TUNEL-positive NPCs (scale bar: 50 μm). The data were presented as the mean ± SD. All the experiments were repeated independently three times. *P < 0.05; **P < 0.01; ***P < 0.001; NS not statistically significant.

There is evidence that DJ-1 promotes the phosphorylation of Akt^37^, and HK2 contains an Akt phosphorylation site^38^. To verify whether phosphorylated Akt links DJ-1 to HK2, western blot analysis of cytosolic and mitochondrial fractions was performed to evaluate the distribution of Akt and phosphorylated Akt. As shown in Fig. 7a–e, overexpression of DJ-1 under oxidative stress conditions increased the level of activated (phosphorylated Ser473) Akt in the cytosol and mitochondria. Notably, overexpression of DJ-1 under oxidative stress conditions increased total Akt levels in the mitochondrial fraction as well, suggesting that activated Akt could promote the mitochondrial distribution of total Akt. In contrast, triciribine (an Akt activation inhibitor) treatment reduced the amount of P-S473-Akt and mitochondrial total Akt. Immunofluorescence colocalization staining further confirmed that overexpression of DJ-1 increased the mitochondrial localization of activated Akt in response to oxidative stress. Similarly, triciribine treatment inhibited the increase in the mitochondrial localization of activated Akt induced by DJ-1 overexpression (Fig. 7f). To investigate the interaction between P-S473-Akt and HK2, proteins from the mitochondrial fraction were coimmunoprecipitated with HK2. The results showed that triciribine treatment reduced the binding of P-S473-Akt to HK2 on mitochondria in DJ-1-overexpressing cells under oxidative stress conditions (Fig. 7g). Furthermore, triciribine treatment reduced the recruitment of Parkin to mitochondria and subsequent mitophagic flux in DJ-1-overexpressing NPCs under oxidative stress conditions (Fig. 7h–l). In addition, triciribine treatment increased the percentage of apoptotic DJ-1-overexpressing NPCs induced by oxidative stress (Fig. 7m, n). Taken together, these data suggest that P-S473-Akt acts as a “bridge” between DJ-1 and HK2.

## Discussion

**Fig. 8:**
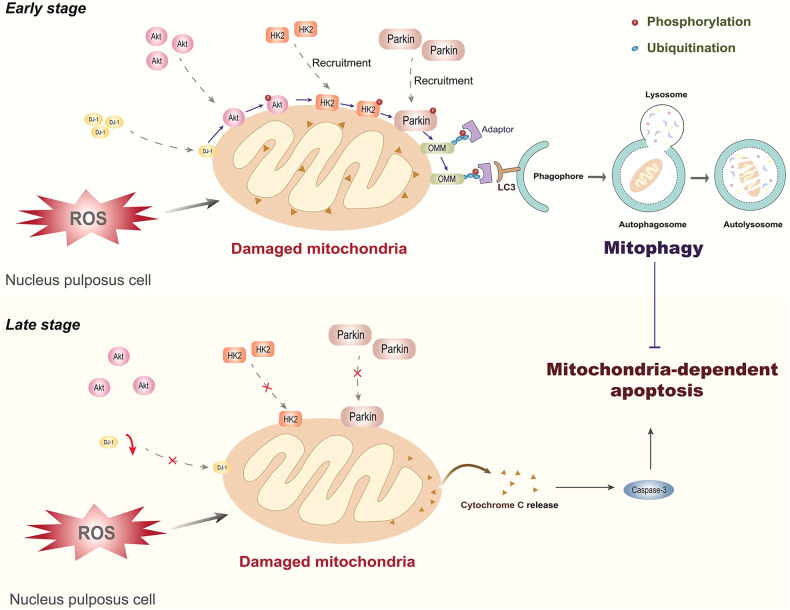
Schematic diagram showing the involvement of DJ-1 in the pathogenesis of IDD. In the early stage of oxidative stress, mitochondrial translocation of endogenous DJ-1 maintains NPC homeostasis by promoting HK2-mediated Parkin-dependent mitophagy through activated Akt; however, in the later stage of oxidative stress, the depletion of cytosolic DJ-1 fails to effectively activate mitophagy in NPCs, leading to Cytochrome c leakage from damaged mitochondria and triggering mitochondria-dependent apoptosis in NPCs. OMM, outer mitochondrial membrane.

This study was the first to show that DJ-1 protects NPCs against mitochondria-dependent apoptosis and ameliorates IDD in vivo by activating HK2-mediated Parkin-dependent mitophagy in response to oxidative stress. Based on our findings, we propose that during the early stage of oxidative stress, endogenous DJ-1 in NPCs translocates from the cytosol to mitochondria and maintains NPC homeostasis by promoting mitochondrial recruitment of HK2 and subsequent Parkin-dependent mitophagy through the phosphorylation of Akt; however, during the late stage of oxidative stress, the depletion of cytosolic DJ-1 prevents mitophagy from being efficiently activated in NPCs, leading to Cytochrome c release from damaged mitochondria to the cytosol and triggering mitochondria-dependent apoptosis (Fig. 8), which manifests as IDD progression in vivo.

NPCs reside in a physiologically avascular and hypoxic tissue niche^39,40^. It is generally believed that NPCs contain only small amounts of mitochondria and rely primarily on the glycolytic pathway for energy. For this reason, mitochondria are thought to possibly play a limited role in intervertebral disc physiology. However, other scholars hold the opposite view. Currently, there is controversy regarding the significance of mitochondria in the metabolism of NPCs within the intervertebral disc. A recent study showed that a large number of mitochondria are indeed present in NPCs; these mitochondria are typically tubular, highly networked, and hypoxia-responsive^41^. Moreover, a growing body of evidence demonstrates that mitochondrial dysfunction leads to excessive death in NPCs through mechanisms such as apoptosis and ferroptosis^42–44^. Consequently, keeping mitochondrial health is essential for maintaining the physiological function of NPCs.

Mitophagy, which is characterized by the selective degradation of damaged mitochondria, is an evolutionarily conserved process that maintains cellular homeostasis by controlling mitochondrial quantity and quality under stress conditions^45,46^. Previous studies have demonstrated the protective effects of moderate mitophagy promotion on NPCs under oxidative stress conditions^15,31,32^. However, the molecular mechanisms regulating mitophagy in NPCs are poorly understood. In the present study, we found that overexpression of DJ-1 promoted Parkin-dependent mitophagy by recruiting HK2 to aggregate towards mitochondria. Indeed, whether DJ-1 is involved in Parkin-dependent mitophagy remains controversial. A previous study reported that DJ-1 could exert mitochondrial protective effects through a pathway parallel to the PINK1/parkin pathway^47^. However, the results of this study^47^ and others^48^ do not exclude DJ-1 as an upstream factor of the PINK1/Parkin pathway because Parkin can rescue both PINK1- and DJ-1-deficient mitochondrial phenotypes, whereas DJ-1 is unable to rescue PINK1-deficient mitochondrial phenotypes. More importantly, the evidence used to indirectly support the parallel role of DJ-1 with the PINK1/Parkin pathway is that these three proteins did not form an active complex, which can be explained by the fact that HK2 mediates the association of DJ-1 with Parkin. A recent study found that DJ-1 can also act as a downstream mediator of PINK1/Parkin-dependent mitophagy^24^. Nevertheless, the findings of our study enrich the body of knowledge on the role of DJ-1 in mitophagy.

HK2 catalyzes the conversion of glucose to glucose-6-phosphate and is the rate-limiting enzyme of the glycolytic pathway. Notably, it has been shown that HK2 responds to mitochondrial depolarization by binding to VDAC and is a substrate for ubiquitination by Parkin^49–51^. In contrast, the dissociation of HK2 from VDAC inhibits ubiquitin phosphorylation and subsequent Parkin-dependent mitophagy^49^. Furthermore, the landscape of a “mitochondrial-autophagosome synapse” during Parkin-dependent mitophagy revealed that HK2 plays a critical role in promoting the assembly of PINK1-Parkin high molecular weight complexes and the phosphorylation of ubiquitin in response to mitochondrial damage^35^. Most importantly, McCoy et al. reported that HK2 activity was essential for the recruitment of Parkin to depolarized mitochondria^52^. These findings were also confirmed in our study, and knockdown of HK2 or blockade of HK2 translocation to mitochondria inhibited the recruitment of Parkin and subsequent mitophagic flux in damaged mitochondria under oxidative stress conditions (Fig. 5c–m, p, q). Interestingly, when DJ-1 expression was suppressed, even HK2 overexpression did not effectively activate mitophagy (Fig. 5n, o, r–u), suggesting that DJ-1 translocation to mitochondria may be a prerequisite for HK2 recruitment to mitochondria.

DJ-1 promotes the activation (phosphorylation) of Akt in response to oxidative stress^37^. We analyzed proteins in the cytosolic and mitochondrial fractions and obtained similar results (Fig. 7a–e). Moreover, there is evidence that HK2 contains a conserved phosphorylation site for Akt (consensus sequence; RXRXXS/T), which contains a threonine at position 473 (Thr473)^38^. Phosphorylation of HK2 by Akt at Thr473 promotes the recruitment of HK2 to the outer mitochondrial membrane^38,53,54^. Collectively, these findings suggest that activated Akt may link DJ-1 and HK2. The results of the present study also confirmed this hypothesis (Fig. 7). However, we are currently uncertain whether DJ-1 directly or indirectly activates Akt. In fact, we only observed the endpoint at which Akt was activated. Furthermore, whether Akt is activated by DJ-1 in the cytosol or mitochondria is unclear. Based on the results that DJ-1 overexpression promotes the mitochondrial translocation of total Akt under oxidative stress conditions and that this process is suppressed by an inhibitor of Akt activation (Fig. 7a, d), we currently prefer to propose that Akt is translocated to mitochondria before being activated by DJ-1 and that activated Akt further phosphorylates the T473 site of HK2. However, the exact molecular mechanism involved remains to be elucidated.

There are several limitations of this study. First, NPCs are exposed to a hypoxic environment under physiological conditions. However, a standard cell culture environment was used for NPC culture in this study, and so the results obtained may not be fully representative of actual conditions in vivo. Second, this study only focused on the effect of DJ-1 on NPCs, and it is not clear whether DJ-1 affects AF and CEP cells. Hence, additional experiments on AF and CEP are needed to truly explain the role of DJ-1 in IDD. Finally, the puncture-induced IDD model cannot simulate the effects of mechanical stress well. Thus, a more appropriate animal model of IDD should be used for validation in future studies.

In conclusion, we found that DJ-1 translocates to mitochondria in response to oxidative stress and activates Parkin-dependent mitophagy by activating Akt to promote mitochondrial recruitment of HK2. Overexpression of DJ-1 can protect NPCs against mitochondria-dependent apoptosis through mitophagy induced by the indicated pathway, which is particularly important in the late stage of oxidative stress when endogenous DJ-1 is depleted. These results provide insight into the protective mechanisms of DJ-1 for NPCs in IDD and suggest that DJ-1 is a promising target for IDD therapy.

### Supplementary information

Supplementary materials
