# Supplementary material for: Involvement of DJ-1 in the pathogenesis of intervertebral disc degeneration via hexokinase 2-mediated mitophagy
Source: Exp Mol Med. 2024 Mar 27;56(3):747–59. doi: 10.1038/s12276-024-01196-0 (PMC10984922; doi:10.1038/s12276-024-01196-0)
Supplement: Supplementary file 1 — Supplementary materials [file 12276_2024_1196_MOESM1_ESM.pdf]

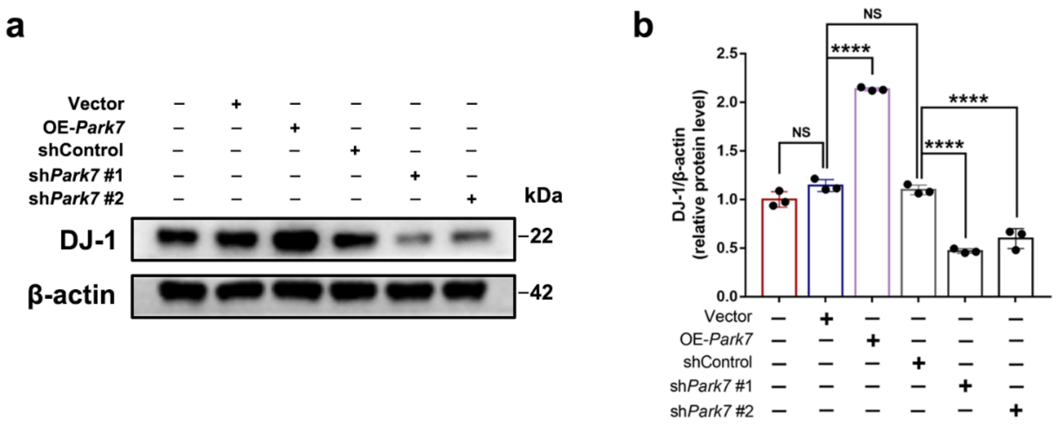

Supplementary Fig. 1. Validation of successful overexpression and knockdown of DJ-1 in NPCs. **a.** Western blot of DJ-1 in NPCs after lentiviral transfection for overexpression or knockdown. **b.** Corresponding densitometric quantification of DJ-1. Quantitative data are presented as mean ± SD. All experiments were repeated three times independently. ****, *P* <0.0001; NS, not statistically significant.

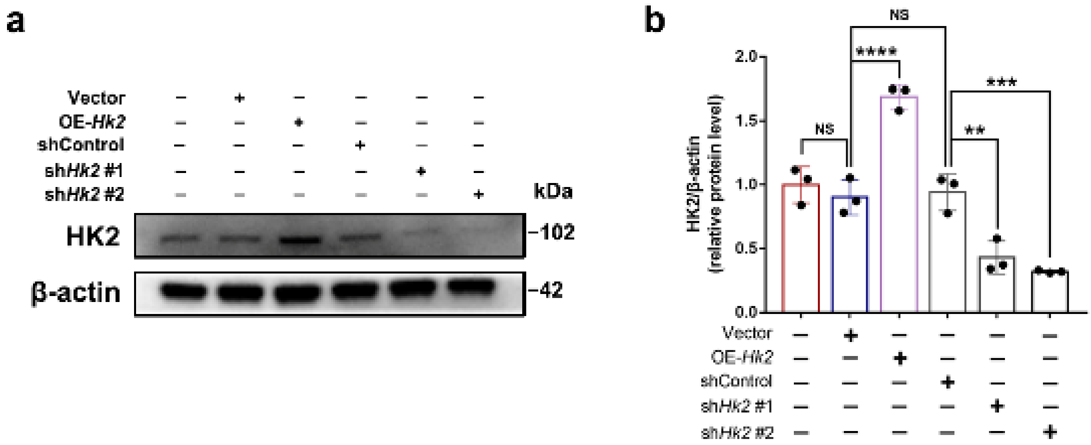

Supplementary Fig. 2. Validation of successful overexpression and knockdown of HK2 in NPCs. **a.** Western blot of HK2 in NPCs after lentiviral transfection for overexpression or knockdown. **b.** Corresponding densitometric quantification of HK2. Quantitative data are presented as mean ± SD. All experiments were repeated three times independently. **, *P* <0.01; ***, *P* <0.001; ****, *P* <0.0001; NS, not statistically significant.
